# Mummification of Pulp Tissue

**Published:** 1900-12-15

**Authors:** H. Prinz


### MUMMIFICATION OF PULP TISSUE.

BY H. PRINZ, D.D.S.

*After the devitalization of a pulp all available efforts should be made by the conscientious practitioner to remove as much of the pulp tissue as possible!* This point I wish to especially emphasize. Even if we are now able to entirely mummify a pulp, this method should not be practiced indiscriminately, as it is antagonistic to one of the fundamental laws of surgery, which teaches us to remove all necrosed tissue, and further, it may create a tendency to carelessness and imperfect work on the part of operator. Therefore, again let me state, that only in such cases as it is utterly impossible to remove all the pulp tissue is application of the mummifying principle justified.

*Mode of application:*—Apply arsenious acid to the exposed pulp in the usual manner. The dressing should be left undisturbed for at least four or five days. By this time the devitalization is more complete, and the dental fibrillæ and odontoblasts which form the junction between dentin and pulp are more disintegrated and therefore facilitate removal of the whole pulp at once. It is advisable to place a small quantity of the mummifying paste upon the devitalized pulp for one or two days. By now removing the pulp one will be often greatly surprised to find it to come away entire, "hard and stiff as a fiddle-string." But in such cases where we have tried in vain to remove all of the pulp tissue, we enlarge the pulp chamber and the canals to about one-third their length. The cavity is then washed with a solution consisting of one part formaldehyd to two parts of water, making about a 10 percent solution. With the warm-air blast we try to saturate the pulp stump thoroughly. This formaldehyd bath, as Boennecken calls it, is very essential. By the great penetrating power of formaldehyd the whole stump will be impregnated and any products of decomposition present at once rendered neutral. If the pulp shows any sign of life it will react immediately, but surrenders soon, as formaldehyd acts practically similar to arsenic as a caustic. The formaldehyd solution should be prepared fresh every four weeks and should be kept in amber-colored bottles. After this preliminary step, the paste proper is applied with sterilized smooth broaches, the canals and crown portion are filled with it, a disk of asbestos felt, previously sterilized in the flame, placed over same, and the whole covered with cement. The filling can be completed at same sitting with whatever material is indicated for the case.

About five years ago I made my first experiments in regard to pulp mummification. At that time glowing reports were published about the wonderful antiseptic properties of formaldehyd, using Soderberg's alum paste as a basis. I substituted at first the liquid and later on the dry formaldehyd for the alum, on the ground that this drug is a superior tanning agent and at the same time possesses a remarkably strong germicidal power. If animal tissue is brought in contact with formaldehyd the albuminoids are changed to an insoluble compound—they are tanned. Tissues shrink but very little or not at all if preserved in such solutions. This is a most important point in regard to the pulp stumps left in the roots, for they keep the foramina hermetically sealed. Formaldehyd has a very great penetrating power; it will combine with ammonia compounds which are the result of putrefaction of the nitrogenous material of animal tissue, rendering these compounds inert. The liquid formaldehyd does not preserve its antiseptic power for a long time; the solutions of the gas in water can not be concentrated above 35 or 40 percent. But the dry formaldehyd, known as trioxymethylene or paraformaldehyd (paraform), a polymerized form of this gas, offers a ready substitute. So long as a minute particle of this paraformaldehyd is left in contact with dead animal tissue, the tissue is absolutely sterile, and any micro-organism or infectious product brought within its reach will be promptly rendered inert. The normal temperature of the mouth is sufficient to cause a slow stream of formaldehyd gas to be generated, creating a continuous process of sterilization. Thymol, a part of the original formula of Miller, I have retained. It is also a very strong antiseptic, and being very slowly soluble in water, it has a more lasting effect. It is stated that thymol combined with certain other drugs is used with great success for preserving anatomical specimens. Paraformaldehyd and thymol have not "body" enough to make a suitable paste; zinc oxid, an inert substance, is therefore added, and very little glycerin is needed to give the mixture the proper pasty consistency. Cocain as part of the paste, as suggested by Boennecken, to make the application painless, is unnecessary. Any vitality left in the pulp stump will promptly manifest itself by the formaldehyd bath.

The formula reads now:

℞ Paraformaldehyd, Thymol aa ʒj;

Zinc oxid - - - - - - - ʒij;

Glycerin q. s. to make a stiff paste.

Keep in amber-colored, well-stoppered bottles. This paste will keep any length of time without changing. Occasionally (in warm weather) a drop of glycerin will separate, but it can be drained off.

What will become of the pulp stumps? may be asked. For experimental work I have used the pulps of calves' teeth, as they are more suitable than those of human teeth. These pulps were placed in proper sized glass tubes, one end sealed with wax, and upon the other end the paste was applied, according to the above given directions, and sealed over with wax. Pulps treated with formaldehyd solution alone were very readily penetrated by the liquid—it took only a few hours; while the paste, if used without the solution, would not accomplish this in less than from fifteen to twenty-five days. Hence the importance of the formaldehyd bath. The same experiments were repeated upon recently extracted teeth with undecomposed pulps with similar results. I had occasion to watch similar experiments in the mouth, and the results showed the same gratifying data. The pulps became a semi-elastic mass, resembling somewhat crude rubber, and filling the canals completely. In all experimental cases I have been able to demonstrate the presence of formaldehyd at the very end of the pulp by the aid of Liebermann's phenol test for formaldehyd, which is as follows: Cut off a few millimeters of the apical part of a mummified pulp, place it in a test tube in about 15 c. c. distilled water, heat for a few minutes and add a drop of a very dilute aqueous phenol solution. Then pour this mixture slowly upon concentrated sulfuric acid in a test tube, so as to form a layer. A bright crimson color appears at the zone of contact, indicating the presence of formaldehyd.

Pulp mummification has passed the experimental stage, and although still in its infancy, has gained a place in dental therapeutics which will become more prominent in the near future.—*Extract from article in October Dental Digest.*

---

PROTECTING THE HANDS FROM INFECTION. — Before touching septic cases it is an excellent plan to wash the hands in vinegar or dilute acetic acid. Slight cuts or abrasions, whose presence was not suspected, are thus revealed, and you may better protect yourself.—*International Journal of Surgery.*

---

DR. ROBERT WAKEFIELD, of New York, recommends the subjoined as an anesthetic free from toxic effects or sloughing:

℞ Cocain mur., grns v;
Boracic acid, grns viii;
Ext. Hamametis;
Aqua distil. aa ℥ i.

—*Items of Interest for July.*

